# Rational protein design of *Bacillus* sp. MN chitosanase for altered substrate binding and production of specific chitosan oligomers

**DOI:** 10.1186/s13036-019-0152-9

**Published:** 2019-03-12

**Authors:** David Gercke, Eva K. Regel, Ratna Singh, Bruno M. Moerschbacher

**Affiliations:** University of Muenster, Institute for Biology and Biotechnology of Plants, Schlossplatz 8, 48143 Münster, Germany

**Keywords:** Chitosan, Chito-oligosaccharides, Chitosanase, Glycoside hydrolase, Protein engineering

## Abstract

**Background:**

Partially acetylated chito-oligosaccharides (paCOS) have a variety of potential applications in different fields, but to harness their benefits, pure paCOS or well-defined paCOS mixtures are essential. For example, if one could produce fully acetylated (A_4_) and fully deacetylated (D_4_) tetramers in abundance, all possible variants of tetrameric paCOS could be generated reliably from them. A promising approach for generating defined paCOS is by enzymatic depolymerization of chitosan polymers using chitosanases, since these enzymes’ subsite specificities directly influence the composition of the paCOS produced; however, enzymatic production of e.g. D_4_ is challenging because the substrate is generally hydrolyzed further by most chitosanases. To overcome this, chitosanases could potentially be engineered so that upon hydrolyzing chitosan, they are unable to hydrolyze certain substrates, leaving well-defined oligomers intact in the hydrolysate.

**Results:**

For this purpose, we performed rational protein engineering on the extensively studied GH 8 chitosanase CSN from *Bacillus* sp. MN. By specifically targeting residues with a predicted function in substrate binding, we created new muteins incapable of efficiently hydrolyzing the fully deacetylated tetramer D_4_, and we were able to demonstrate efficient large-scale production of D_4_ with an altered version of CSN. Furthermore, we were able to uncover differences in the substrate positioning and subsite specificities of the muteins, which result in altered paCOS mixtures produced from partially acetylated chitosan polymers, with possibly altered bioactivities.

**Conclusion:**

The value of protein engineering as a tool for the more efficient production of pure oligomers and potentially bioactive paCOS mixtures was demonstrated by the results and the suitability of specific muteins for the large-scale production of strictly defined, pure paCOS in a batch process was shown using the example of D_4_.

**Electronic supplementary material:**

The online version of this article (10.1186/s13036-019-0152-9) contains supplementary material, which is available to authorized users.

## Background

Chitosan, a family of polymers consisting of β-1,4-linked *N*-acetyl-d-glucosamine (GlcNAc or A) and d-glucosamine (GlcN or D) units, is a group of commercially available molecules that are commonly generated by the partial chemical deacetylation of chitin. Producing chitosan is economically feasible since the required chitin is abundant; it is mostly derived from the crustacean shells discarded by the seafood industry [1]. Chitosans are biocompatible, biodegradable, non-toxic, and show a variety of interesting bioactivities, but there is also a growing interest in producing partially acetylated chito-oligosaccharides (paCOS) that can be derived from chitosans by partial depolymerization [1–4]. The oligomers share many of chitosans’ positive properties, but they are additionally soluble at a neutral pH and have a lower viscosity, which is beneficial for their use in many areas, such as foliar application in agriculture [5]. In fact, paCOS produced by naturally occurring hydrolases might be responsible for some effects of chitosans in plant protection and wound healing [2, 6]. Studies have found possible applications for paCOS in medicine, cosmetics, waste water treatment, and agriculture based on, e.g., their antimicrobial, antitumor, and immune-regulatory effects, but paCOS can also act as elicitors that induce various defense responses in plants or prime plants by inducing a state of enhanced defense in which they are more resistant to subsequent abiotic or biotic stresses [5, 7–10].

However, in some cases the reproducibility of paCOS bioactivities is problematic, as many studies have used poorly defined mixtures of paCOS varying in their degree of polymerization (DP), degree of acetylation (DA), and pattern of acetylation (PA) [2]. Just as it has been described for chitosan polymers, the DP and DA of paCOS are known to directly affect their bioactivities [11–14]; the PA’s influence on paCOS bioactivities has been generally assumed, but only recently have studies started to decipher this [9, 15, 16]. Further, recent advances have now made it possible to fully characterize paCOS mixtures via quantitative mass spectrometric sequencing [17], but the major hurdle of reproducibly generating suitable paCOS mixtures or pure paCOS remains, rendering it difficult to determine how the PA specifically affects bioactivity. In this context, Hembach et al. recently achieved small-scale generation of all 14 possible tetrameric paCOS from the fully acetylated A_4_ and the fully deacetylated D_4_ [18]. While both substrates are already commercially available, they are extremely expensive and larger quantities would be required to allow bioactivity tests that could potentially give insights into the effect of the PA.

One promising approach to produce more defined paCOS involves using endo-acting chitosanases (E.C. 3.2.1.132) to depolymerize chitosans: As the process is enzymatic, it can be performed under mild conditions, is easy to control, and can potentially yield high amounts of oligomers with large DP. Chitosanases can be subdivided into families based on their amino acid sequences and into classes based on their subsite specificities [19, 20]. Four classes had previously been defined in this context. Class II enzymes are only able to cleave GlcN-GlcN linkages. Class I enzymes can additionally cleave GlcNAc-GlcN bonds and class III enzymes are capable of cleaving GlcN-GlcN and GlcN-GlcNAc linkages. The enzymes from class IV can cleave all bonds except the GlcNAc-GlcNAc bond that usually only chitinases can cleave [20, 21]. More recently, a new classification system was proposed based not only on the specificities of the enzymes’ subsites (− 1) and (+ 1) directly adjacent to the hydrolyzed bond, but also on the subsites (− 2) and (+ 2), taking into account not only absolute specificities but also relative preferences [22]. Subsite specificities and preferences of chitosanases for deacetylated or acetylated units are relevant for paCOS production because the composition of the generated oligomer mixtures is defined by them. Another important characteristic of chitosanases is the minimum DP of the end products they generate since oligomers with a higher DP often show stronger bioactivities [11, 12, 23, 24].

Chitosanases generally show lower hydrolytic efficiency on smaller substrates when not all subsites are occupied and accordingly, these oligomers accumulate during hydrolysis and can be purified. However, if the enzyme is capable of hydrolyzing the desired oligosaccharide product, the yield will be decreased as a result. Degradation of the product can be reduced by utilizing ultrafiltration (UF) membranes to separate enzyme and products. Experimental setups involving the use of UF membranes have been successfully applied to produce chitosan oligosaccharides in the past [25, 26]. While effective, applying such setups involves higher initial investments and difficulties such as membrane fouling might occur [27]. Applying an enzyme incapable of hydrolyzing D_4_ would allow an effective and cheap production of the tetramer in a simple batch process. To find a suitable chitosanase for such an approach, one option is to identify and characterize naturally occurring enzymes for their ability to produce specific paCOS; alternatively, another option is to use rational protein design to alter already well-characterized enzymes.

The latter approach was chosen in this study because we wanted to obtain an enzyme unable to hydrolyze D_4_, a substrate that is normally readily hydrolysable by bacterial chitosanases [28–31]. Accordingly, the main products from low DA chitosan polymer are often D_2_ and D_3_ [32]. We performed engineering on a well-characterized, efficiently expressed enzyme with high activity: The chitosanase CSN from *Bacillus* sp. MN (*Bsp*CsnMN, GenBank accession no. JQ425408), subsequently referred to as CSN [33]. This enzyme can be classified as GH 8, former class III and current class A [22]. CSN contains seven subsites in its active center [34], and functional roles for a variety of residues have been identified [35]. Substrate binding first involves the substrate forming a “V-shape” conformation before hydrolysis, a process that is aided by the residues E59, W118, and Y270, among others [34, 36]. Therefore, we attempted to abolish the activity of CSN on the fully deacetylated chitosan tetramer D_4_ by disrupting substrate binding at specific subsites. As D_4_ is readily accepted as a substrate and represents the smallest oligomer CSN can hydrolyze, any resulting muteins unable to cleave D_4_ could be used to produce high quantities of the oligomer which, together with fully acetylated A_4_, can serve as a starting material for the production of all partially acetylated chitosan tetramers at a larger scale [18]. Moreover, the hydrolysates produced with the muteins when acting on partially acetylated chitosan polymers are expected to differ from those produced with CSN wildtype, thus widening the spectrum of producible paCOS mixtures that could potentially also show altered bioactivities.

## Results

**Fig. 1:**
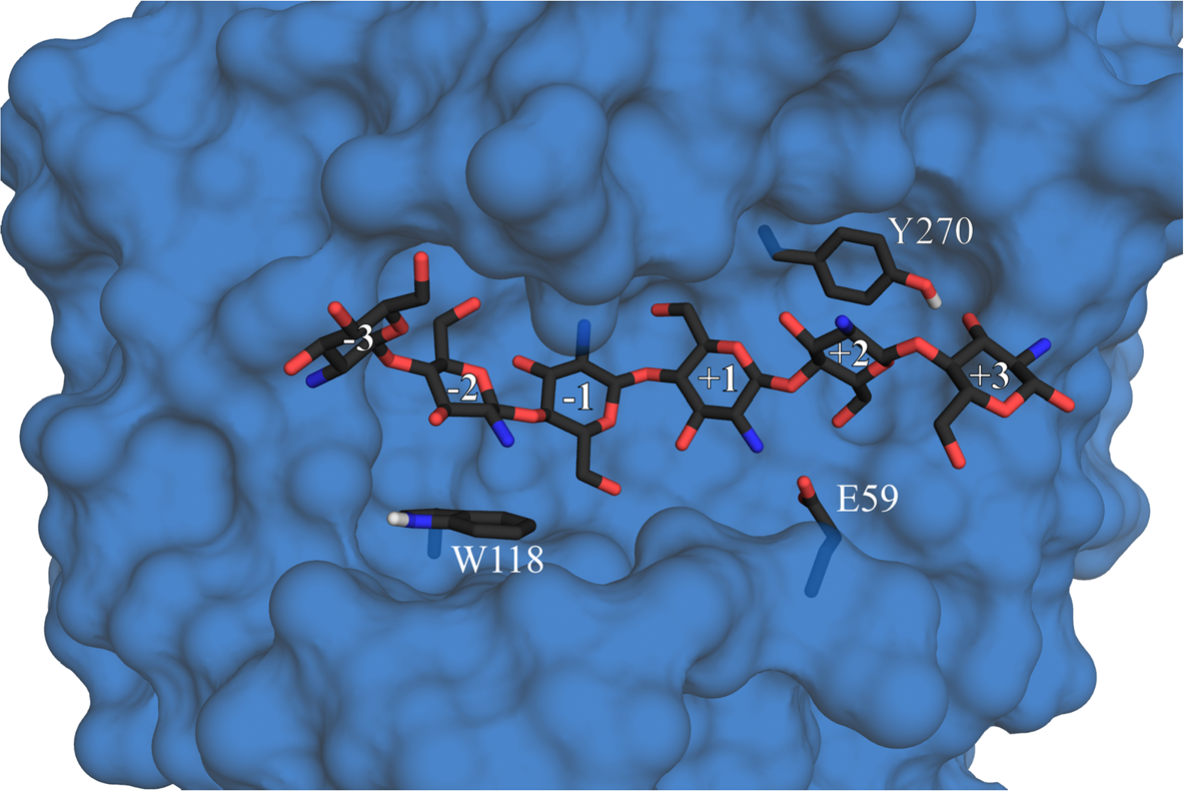
Selected CSN residues with a putative function in substrate binding. A three-dimensional homology-based model of CSN based on the crystal structure of *Bacillus* sp. K17 chitosanase (PDB: 1V5C, amino acid sequence identity: 97.47%), illustrating the side chain positions of the amino acids W118, E59, and Y270 relative to the docked substrate D_6_. The subsites (− 3) to (+ 3) are indicated. The enzyme surface without the side chains of the listed amino acids is pictured in blue, the labeled amino acid side chains emerging from the surface and D_6_ are colored by element.

To obtain muteins of CSN that display reduced activity on D_4_ but retain high activity on longer substrates, we generated CSN-E59A, CSN-W118A, and CSN-Y270A. In the active center of CSN, D_4_ is forced into a “V-shape” conformation and is symmetrically positioned between the subsites (− 2) and (+ 2) before hydrolysis [35, 36]. By disrupting the substrate binding at either subsite (− 2) or subsite (+ 2), the formation of the “V-shape” and a subsequent cleavage of the substrate should no longer be possible. Nonetheless, such mutations should not prevent activity on longer substrates with a DP of 5 and above, because these substrates can occupy additional subsites beyond (− 2) and (+ 2) that could potentially stabilize their binding and enable formation of the correct substrate conformation and subsequent hydrolysis. For one mutein, since residue W118 forms stacking interactions with a sugar unit at subsite (− 2) (Fig. 1) [34], we attempted to disrupt stable substrate binding from subsites (− 2) to (+ 2) by changing the tryptophan at position 118 to alanine. For the resulting CSN-W118A, occupation of the subsite (− 3) could potentially stabilize binding of D_5_ or longer oligomers and allow efficient cleavage. Analogously, we created another two muteins by changing either Y270 at subsite (+ 2) or E59, positioned between subsites (+ 1) and (+ 2), to alanine. In these cases, D_5_ should still be able to occupy subsite (+ 3) and achieve stabilized binding. CSN and the muteins were purified using *Strep*-tag II/*Strep*-Tactin affinity chromatography and analyzed via western immunoblotting (Additional file 1: Figure S1).

### Hydrolytic efficiency towards chitosan and COS

**Fig. 2:**
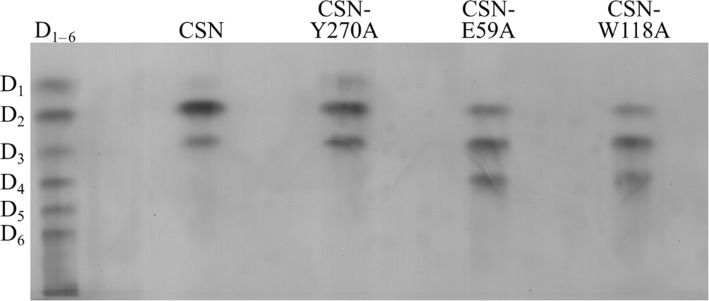
Hydrolysates of CSN, CSN-Y270A, CSN-E59A, and CSN-W118A with chitosan polymer DA 1.5% as a substrate. Hydrolysates were produced by incubating 1 mg/ml chitosan polymer DA 1.5% and each enzyme (0.05 μM of CSN, 0.42 μM of CSN-Y270A, 0.05 μM of CSN-E59A, and 2.5 μM of CSN-W118A) for 16 h at 37 °C and then used for a TLC. A mixture of D_1–6_ was utilized as a standard

To check the muteins for a decreased activity on D_4_, we initially compared the product profiles from a low DA chitosan polymer (Fig. 2). As expected from previous studies, hydrolysates produced with CSN wildtype contained mainly D_2_ and D_3_ as end products. For both CSN-E59A and CSN-W118A, D_4_ was present in addition to D_2_ and D_3_, already indicating a reduced activity on the tetramer. For CSN-Y270A, the exchange of Y270 to alanine did not seem to disrupt the hydrolytic activity on D_4_, as this oligomer was not present in the hydrolysate; rather, increased amounts of D_1_ and D_3_ were detectable.

**Fig. 3:**
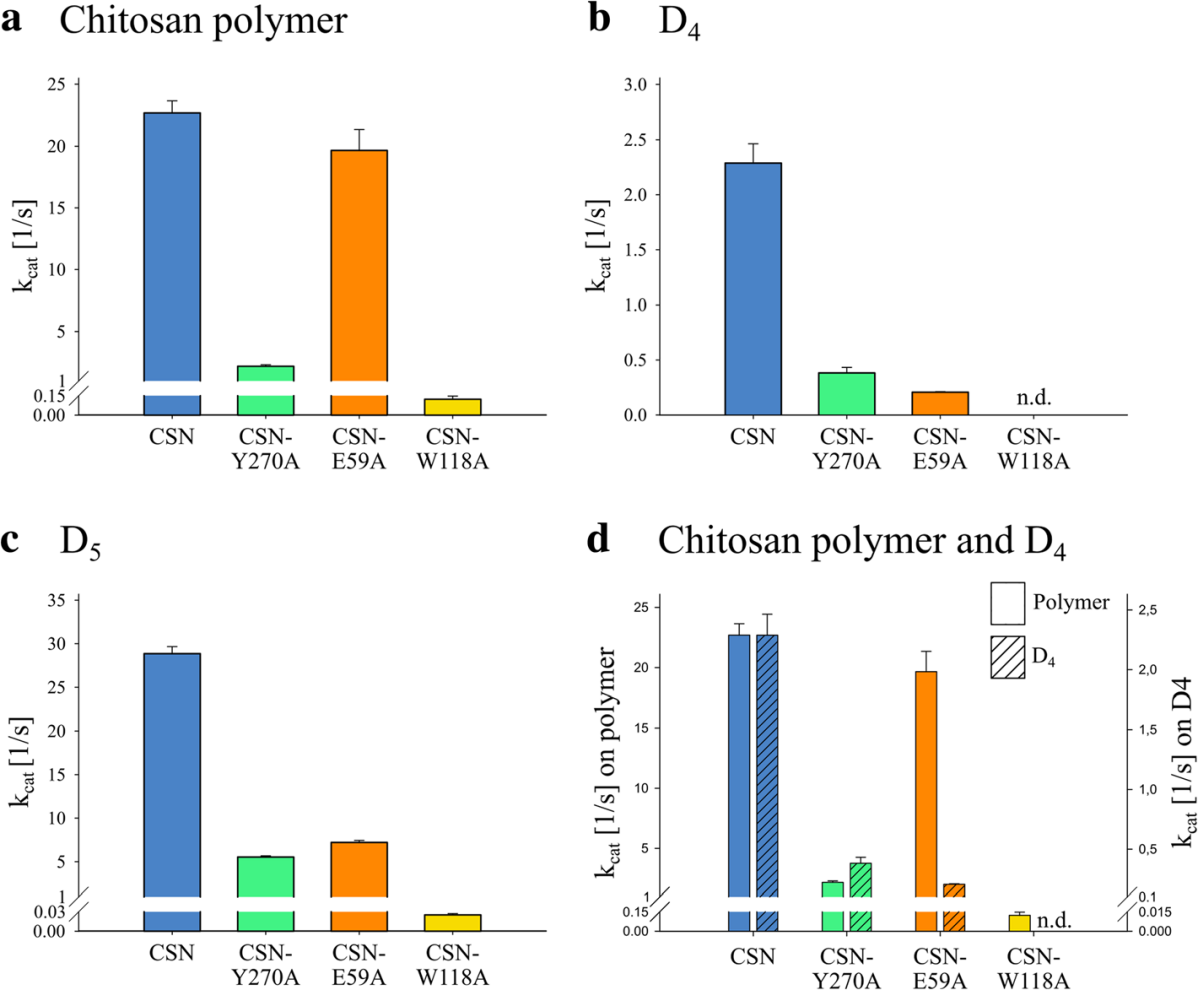
Kinetic parameters of CSN, CSN-Y270A, CSN-E59A, and CSN-W118A on different substrates. The catalytic rate constant k_cat_ at 30 °C is illustrated for the substrates (**a**) chitosan polymer DA 1.5%, (**b**) D_4_, and (**c**) D_5_. Also, (**d**) a direct comparison between chitosan polymer DA 1.5% and D_4_ is given. Three independent enzyme batches were used for each enzyme, and the kinetics were performed as triplicates for each batch. Data given are the mean values of all nine replicates, and the standard deviations between the three independent enzyme batches are indicated. A 30 min incubation period was used for CSN-E59A for D_4_ and for CSN-W118A. All other reactions were incubated for 10 min. Different enzyme concentrations were used for chitosan polymer DA 1.5% (0.05 μM CSN, 0.3 μM CSN-Y270A, 0.05 μM CSN-E59A, and 2.5 μM CSN-W118A), D_4_ (0.04 μM CSN, 0.023 μM CSN-Y270A, and 0.1 μM CSN-E59A), and D_5_ (0.02 μM CSN, 0.168 μM CSN-Y270A, 0.2 μM CSN-E59A, and 1 μM CSN-W118A)

Kinetics on chitosan polymer DA 1.5% as well as on the oligomers D_4_ and D_5_ were recorded to verify these first findings (Fig. 3). CSN showed high activity on the polymer, and it was even slightly more active on D_5_. By comparison, the tetramer D_4_ proved to be a poor substrate. The obtained value of 2.3 s^− 1^ for the catalytic rate constant (k_cat_) on D_4_ was ca. tenfold lower than for the other two substrates, but efficient hydrolysis of D_4_ was still possible. Compared to CSN, the mutein CSN-Y270A showed a pronounced, consistently lower activity, where for all tested substrates, the k_cat_ was reduced by a factor of around 10. For CSN-E59A, the activity on the polymer was only barely affected by the substitution, such that when hydrolyzing the polymer, this mutein retained about 87% of the maximum reaction velocity of CSN. However, CSN-E59A did have a drastically reduced activity on D_4_; compared to the k_cat_ of CSN on D_4_, the k_cat_ of CSN-E59A on D_4_ was nearly 10 times lower, and it was only about 1% of the k_cat_ for CSN-E59A on the chitosan polymer (Fig. 3d). Mutein CSN-W118A did show an almost 200-fold decrease in activity on the polymer compared to CSN, but its hydrolytic efficiency on D_4_ was far lower or possibly even abolished entirely, as no activity was detectable, and no kinetics could be determined as a result.

When comparing the catalytic rate constants between the substrates D_4_ and D_5_, all enzymes were multiple times more active when the additional fifth subsite was occupied during substrate binding. CSN and CSN-Y270A showed 12.6-fold and 14.5-fold higher k_cat_ values, respectively, for D_5_ over D_4_, and CSN-E59A showed an even more drastic effect, with a 34.8-fold increase for D_5_ over D_4_. For CSN-W118A, which showed no activity at all on D_4_, the hydrolysis of D_5_ did occur, albeit only very slowly.

### In-depth characterization of substrate binding with deacetylated substrates

**Fig. 4:**
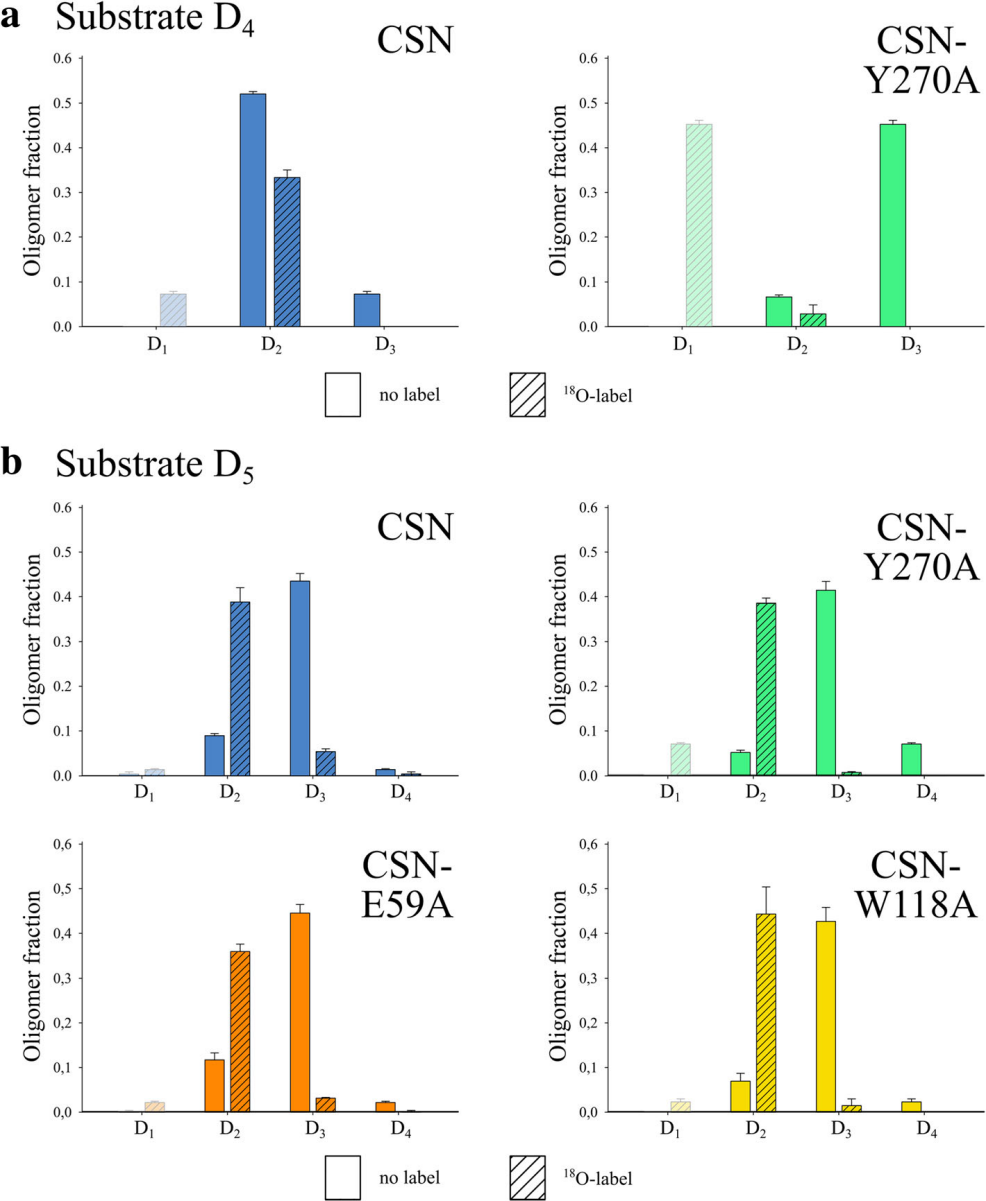
Cleavage position when digesting (**a**) D4 and (**b**) D5 with CSN, CSN-Y270A, CSN-E59A, and CSN-W118A. The enzymes were incubated with 1 mM of ^18^O-labeled D_4_ or D_5_ at 30 °C for 10 min and the products were immediately measured using UHPLC-ELSD-ESI-MS^1^. The enzyme concentrations when using D_4_ were 0.02 μM for CSN and 0.035 μM for CSN-Y270A. When using D_5_, 0.16 μM of CSN, 0.42 μM of CSN-Y270A, 0.33 μM of CSN-E59A, and 5 μM of CSN-W118A were applied. Quantification of the oligomers with and without the label at the reducing end was done by comparison with external oligomer standards. The amounts of D_1_ (faded bars) were not determined by quantification of D_1_ but instead deduced from the measured amounts of D_3_ or D_4_. The combined amount of all oligomers was set to 1 for each of the enzymes. Three independent enzyme batches were used for each enzyme, and the experiments were performed as triplicates for each batch. Data given are the mean values of all nine replicates and the standard deviations between the three independent enzyme batches are indicated

We successfully generated muteins unable to efficiently hydrolyze D_4_ (CSN-E59A and CSN-W118A), but the results did not allow us to determine how the occupation of a fifth subsite mediated more stable substrate binding. To investigate this and to analyze why CSN-Y270A produced a different oligomer composition than the other two muteins, we performed ^18^O-labeling of the reducing ends of these oligomers. As substrates, we used D_4_ and D_5_ with an ^18^O atom instead of a ^16^O atom incorporated at the reducing end, and the resulting labeled and non-labeled products were measured via UHPLC-ELSD-ESI-MS^1^ to determine the substrate positioning during hydrolysis (Fig. 4). Since D_1_ ionizes very poorly with the experimental setup used, the amounts of the monomer (faded bars in Fig. 4) were not derived from quantification with external standards, but they were instead deduced from the quantified remaining products.

As expected, the main products produced by CSN from D_4_ were labeled and non-labeled D_2_ resulting from positioning the substrate within subsites (− 2) to (+ 2). The slightly differing amounts can be explained by some of the produced oligomers losing their ^18^O label during the incubation. Mutein CSN-Y270A showed different results for D_4_, such that the D_4_ substrate most frequently shifted its positioning to subsites (− 3) to (+ 1), resulting in the production of labeled D_1_ and non-labeled D_3_. This shift was to some extent also visible when using D_5_ as a substrate: While CSN and CSN-Y270A both mainly positioned the oligomer from subsites (− 3) to (+ 2), the mutein also produced non-labeled D_4_ and, presumably, labeled D_1_ which occurred when the substrate occupied subsites (− 3) to (+ 1) while leaving the unit at the non-reducing end unbound. For CSN-E59A and CSN-W118A, both muteins bound the D_5_ substrate from subsite (− 3) to subsite (+ 2), as was seen for CSN.

**Fig. 5:**
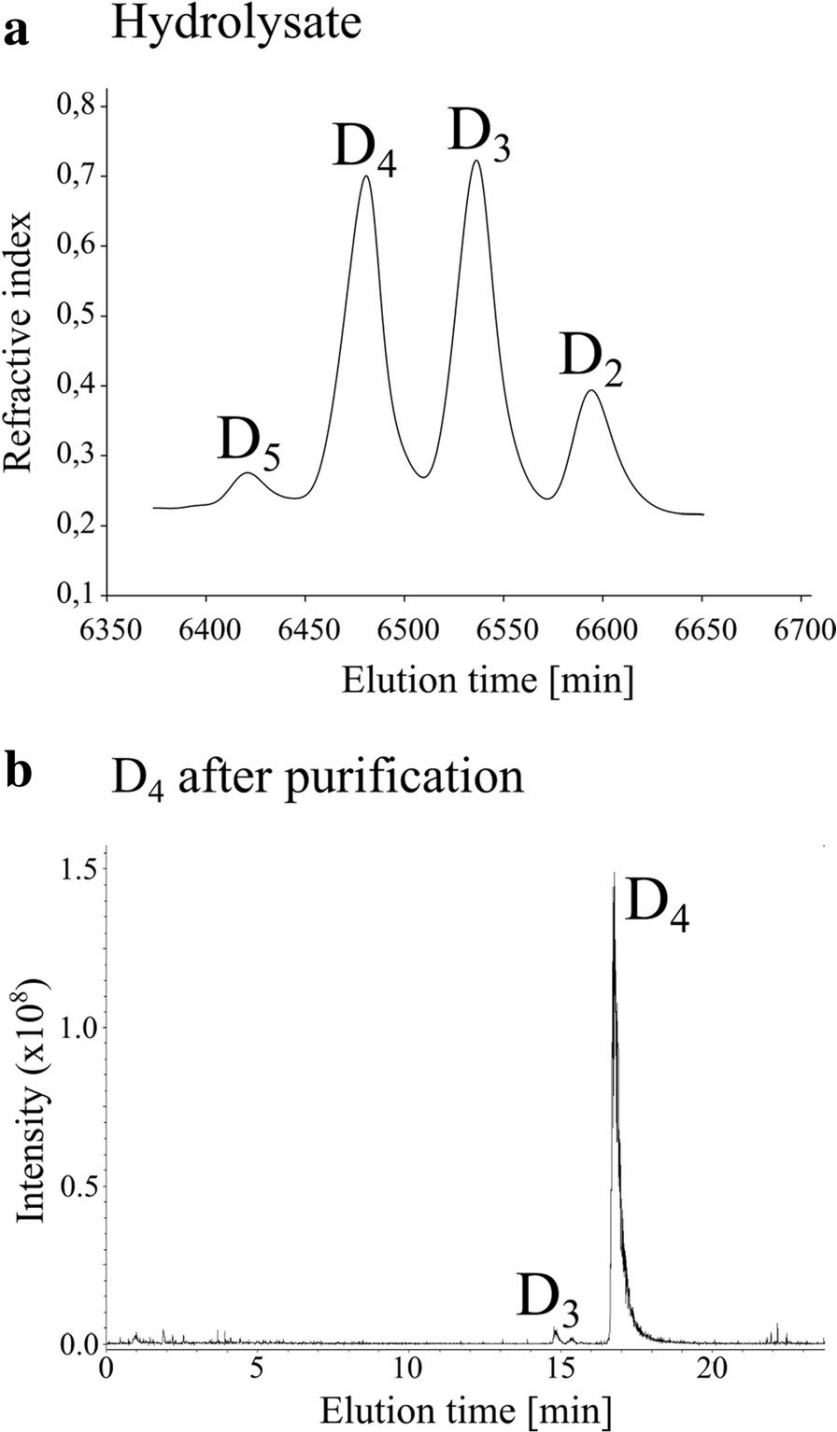
Production of D_4_ with CSN-W118A at a semitechnical scale. One g of chitosan polymer DA 1.5% was hydrolyzed with 0.367 μM of CSN-W118A for 51.5 h at 37 °C. **a** A part of the elution profile for the separation of the chitosan oligomers by SEC. The products were identified using UHPLC-ESI-MS^1^ measurements. Fractions containing D_4_ were combined and washed, and the amount and purity of the product were determined by (**b**) UHPLC-ELSD-ESI-MS^1^ measurements

Hydrolysis of low DA chitosan polymer resulted in the accumulation of D_4_ for both CSN-E59A and CSN-W118A. Therefore, this process, combined with a purification of the oligomers using size-exclusion chromatography, should allow for efficient production of D_4_. Both muteins were tested for this purpose at a semitechnical scale with 1 g of chitosan polymer DA 1.5% as a substrate. The production was found to be possible with both muteins, but it was easier to handle and more efficient with CSN-W118A (Fig. 5). The incubation times were 21 h and 52 h for CSN-E59A and CSN-W118A, respectively, but only 136 mg of D_4_ were purified from the CSN-E59A hydrolysate as compared to more than 400 mg of D_4_, which was virtually free of other reducing sugars, for CSN-W118A.

### Subsite specificities and preferences

**Fig. 6:**
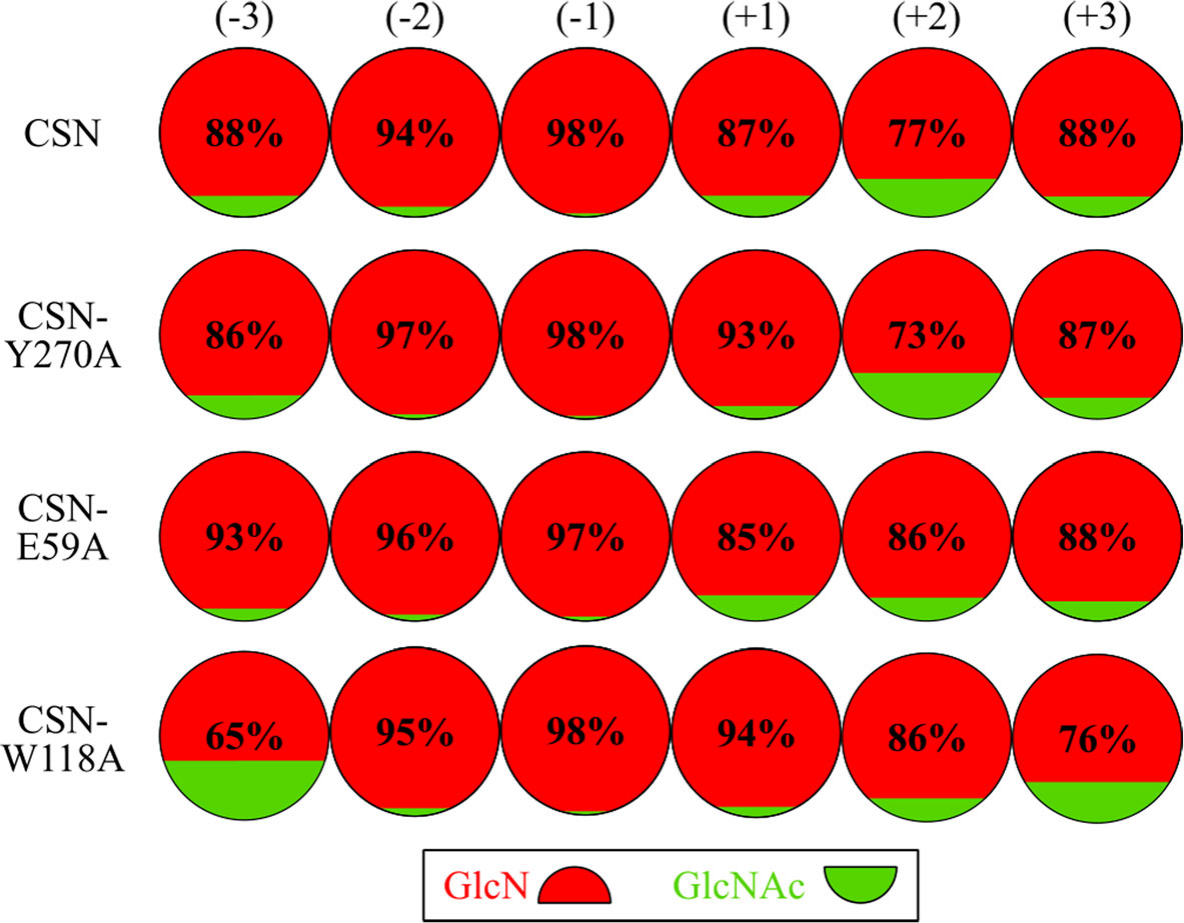
Subsite specificities and preferences of CSN, CSN-Y270A, CSN-E59A, and CSN-W118A on chitosan polymer DA 30%. The enzymes were incubated at 37 °C with 1 mg/ml chitosan polymer DA 30% until the endpoint (60 h in total). CSN, CSN-Y270A, CSN-E59A, and CSN-W118A were used at concentrations of 0.05 μM, 0.3 μM, 0.05 μM, and 2.5 μM, respectively. The frequencies of GlcN and GlcNAc units occurring at and near the reducing and non-reducing ends of the oligomers produced were determined using quantitative mass spectrometric sequencing. The mean values for the GlcN frequencies at the subsites (− 3) to (+ 3) are indicated. The experiments were performed as triplicates with one batch of each enzyme

Using completely deacetylated substrates, distinct differences in the catalytic turnover rate and the substrate positioning were found between CSN and the muteins. In a second step, we compared the more complex and potentially bioactive paCOS product mixtures with higher DP and DA derived from incubating a medium DA substrate with the enzymes. To investigate if the amino acid substitutions changed the subsite specificities of the chitosanase, chitosan polymer DA 30% was hydrolyzed to the endpoint, and the composition of the emerging oligomers was analyzed via quantitative mass spectrometric sequencing (Fig. 6). The subsites (− 3) to (+ 3) were included in the experiments. No drastic differences between CSN and the tested muteins were found, but slight changes were observed. CSN’s known absolute specificity for GlcN at subsites (− 2) and (− 1) and a general preference for GlcN units [22] were determined for all muteins under the conditions tested. Nonetheless, CSN-E59A and CSN-W118A showed a reduced tolerance for the presence of a GlcNAc unit in the substrate at subsite (+ 2), and for both CSN-Y270A and CSN-W118A, subsite (+ 1) was occupied with a GlcN unit more frequently than in the wildtype CSN; CSN-W118A showed an increased tolerance for GlcNAc at subsites (− 3) and, less marked, (+ 3).

**Fig. 7:**
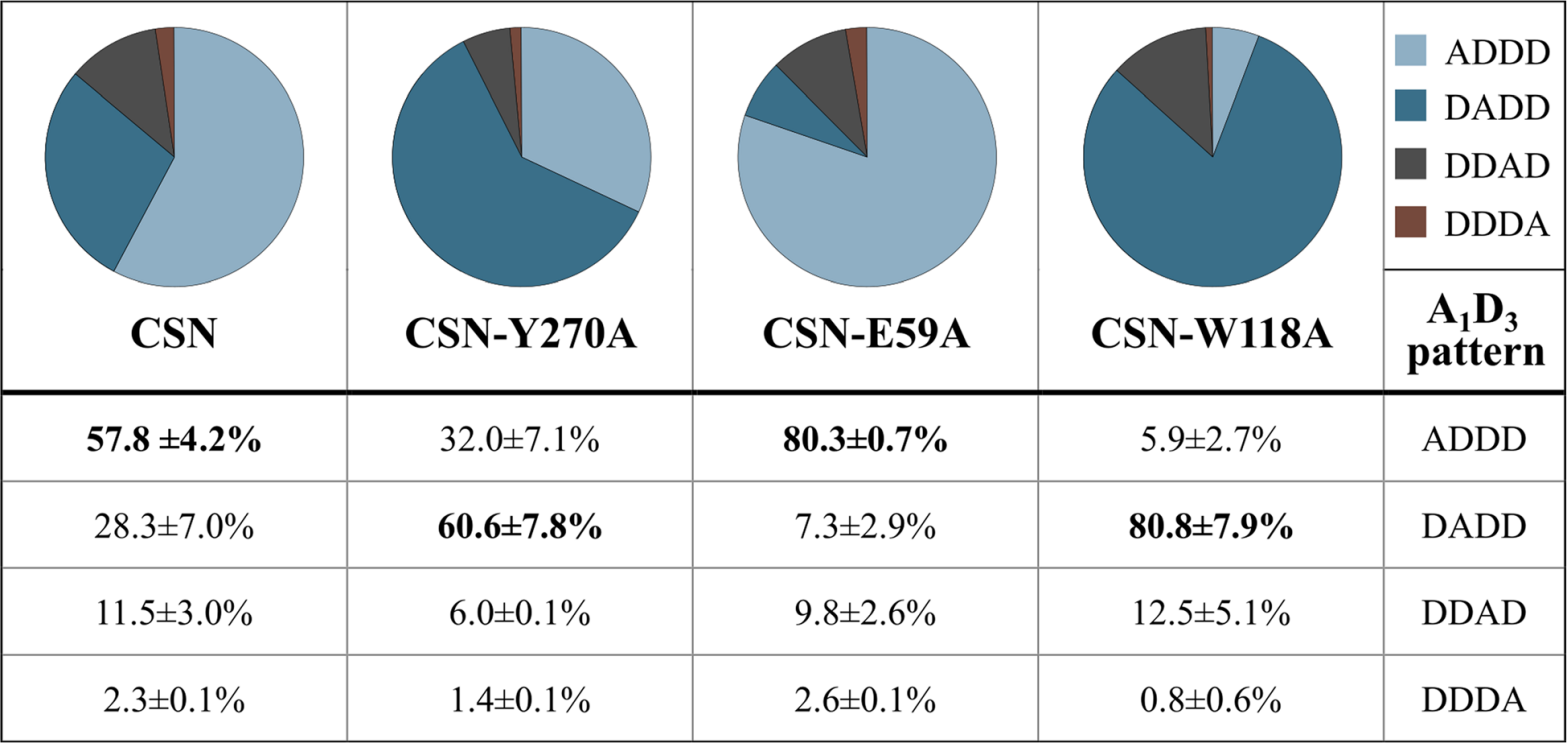
Differences in the pattern of acetylation between CSN, CSN-Y270A, CSN-E59A, and CSN-W118A for A_1_D_3_. The results are obtained from the reactions described in Fig. 6. The most prevalent monoacetylated tetramer product is indicated in bold for each enzyme. A combination of quantitative UHPLC-ELSD-ESI-MS^1^ and UHPLC-ELSD-ESI-MS^2^ measurements was used to determine the absolute amount of A_1_D_3_ in the hydrolysates and the relative frequency of each possible pattern. The experiments were performed as triplicates with one batch of each enzyme, the standard deviation is indicated

Since only minor differences were seen for the GlcN and GlcNAc frequencies in the chitosan polymer DA 30% hydrolysates produced with CSN and the muteins, we next analyzed the data for oligomers with the same DP and DA but different PAs in more detail. The oligomers chosen for this comparison were the monoacetylated tetramers A_1_D_3_. These paCOS were quite abundant, as they constituted ca. 10% (*w*/w) of the hydrolysates (Additional file 1: Figure S2). All four possible PAs were seen for CSN, but by far the most abundant was ADDD, with the GlcNAc unit at the non-reducing end (Fig. 7). The second most frequent PA was DADD, followed by DDAD; only trace amounts of the oligomer DDDA were detectable. Altered ratios for the different PAs were found between CSN and the tested muteins. In contrast to CSN, the most common PA of A_1_D_3_ observed for CSN-Y270A and CSN-W118A was DADD, the difference being most striking for CSN-W118A which produced almost no ADDD. Conversely, CSN-E59A produced even more ADDD than CSN, such that ADDD made up ca. 80% of the A_1_D_3_ in its hydrolysis products. Overall, the slight differences in the preferences for GlcN and the tolerance for GlcNAc at certain subsites found between CSN and the muteins led to more distinct differences in the PAs of the resulting monoacetylated A_1_D_3_ tetramers.

## Discussion

Our concept of performing mutagenesis on CSN to abolish its ability to efficiently hydrolyze D_4_ by targeting specific residues involved in substrate binding was successful for muteins CSN-E59A and CSN-W118A. These two muteins had no or very low activity on the fully deacetylated tetramer (Fig. 3), illustrating that we were able to increase the minimum DP that CSN can efficiently cleave from 4 to 5. The remarkably small loss of activity that CSN-E59A showed toward the polymer indicates that the predicted role of E59 in substrate recognition at subsites (+ 1) and (+ 2) [36] is less relevant when more subsites are occupied. This may be because at the same subsites, other residues such as F365 and N271 (Additional file 1: Figure S3) likely also interact with the substrate, so that binding and hydrolysis of D_4_ can still occur, albeit at a very low rate; these residues have been implicated in such binding in docking studies [34]. Unlike E59, W118 seems to be indispensable for enzyme-substrate interaction at subsite (− 2). This was demonstrated by the mutein’s non-detectable activity on D_4_ and simultaneously low k_cat_ values on both D_5_ and the DA 1.5% chitosan polymer.

The results also allowed for detailed insights into the magnitude of the substrate interactions at the different subsites relative to each other. We predicted that the impaired substrate binding at the mutated subsites would be compensated for when longer substrates were introduced because the oligomers would be able to occupy an additional fifth subsite, thereby allowing the substrate to form the correct “V-shape” conformation. This expected compensation was confirmed by the observations that CSN-E59A showed a stronger increase in activity than CSN when comparing the k_cat_ values on D_4_ and D_5_ and that CSN-W118A had detectable hydrolytic activity on D_5_ but not D_4_ (Fig. 3). For both CSN-E59A and CSN-W118A, the binding of D_5_ involved subsites (− 2) to (+ 2) but it also involved the fifth subsite, (− 3) (Fig. 4). This was contrary to the initial prediction for CSN-E59A and indicates that CSN much more strongly interacts with the substrate at subsite (− 3) than at subsite (+ 3), which was virtually never occupied when binding D_5_ (Additional file 1: Figure S4).

Moreover, CSN-Y270A showed an unexpected shift in substrate positioning towards the minus subsites when binding D_5_, but especially when binding D_4_ (observed via ^18^O-labeled substrates). The resulting generation of D_1_ was highly uncommon for an endo-acting chitosanase (EC 3.2.1.132), but it has been reported before for a few fungal enzymes [37, 38]. This shift indicates that the loss-of-function mutation on Y270 cannot be fully compensated for by other residues at this subsite. When only binding four GlcN units, positioning the substrate at subsites (− 3) to (+ 1) is energetically more favorable than positioning them at subsites (− 2) to (+ 2), since it allows the enzyme to interact using four subsites that are not directly affected by the mutation. This observation additionally highlights that stronger interactions occur at subsite (− 3) as compared to subsite (+ 3). One residue that is predicted to predominantly contribute to these strong interactions at subsite (− 3) is W187, which forms stacking interactions [34] (Additional file 1: Figure S3).

Interestingly, positioning of the D_4_ substrate was not shifted for the other two muteins. In CSN-W118A, the mutation at subsite (− 2) might have shifted D_4_ binding to subsites (− 1) to (+ 3). The reason this shift did not happen is likely because the relatively weak interactions at subsite (+ 3) prevented any shift of D_4_ towards the plus subsites from being more energetically favorable. In the case of CSN-E59A, the original E59 residue likely helps the substrate take the “V-shape” conformation by creating two hydrogen bonds with the amino group of GlcN unit at subsite (+ 1) and the C6-hydroxy group at subsite (+ 2) [36]; therefore, mutating this residue would still impair binding at subsite (+ 1), even if D_4_ shifted to subsites (− 3) to (+ 1).

One practical application for the generated muteins CSN-E59A and CSN-W118A is that they can be utilized in a simple batch process to produce D_4_ in abundance (Fig. 5); the resulting D_4_ can be used as a substrate for the production of pure, fully defined paCOS tetramers using chitin deacetylases in reverse catalysis [18]. The fully acetylated tetramer A_4_ was recently shown to modulate the expression of genes involved in development, vegetative growth, and carbon and nitrogen metabolism in *Arabidopsis thaliana* [39], but tetrameric paCOS were not yet tested. The ability to enzymatically produce all fourteen partially acetylated chitosan tetramers [18] in principle allows for the rigorous testing of the influence of PA on the bioactivities of paCOS, but rather large amounts of pure D_4_ and A_4_ are required to upscale tetrameric paCOS production. Establishing an efficient and easy means of producing D_4_, which can easily be converted into the fully acetylated chitin tetramer A_4_ using chemical *N*-acetylation [40], represents a critical step in this endeavor.

While producing D_4_ using CSN-E59A was not particularly efficient, giving a yield of under 15% (*w*/w), CSN-W118A yielded more than 400 mg of D_4_ from 1 g of chitosan polymer, and the D_4_ was virtually free of other reducing sugars. While we had to use a higher concentration of CSN-W118A compared to CSN-E59A, the former was clearly more suitable for efficient production of D_4_. Using a different approach for engineering, Regel et al. recently published the generation of a mutein of CSN named CSN-VRE which exhibited a strongly altered subsite specificity [35]. This enzyme could be utilized to produce oligomer mixtures containing D_4_ as well, but a production of pure D_4_ at a larger scale was not demonstrated. Moreover, CSN-VRE showed a drastically lower catalytic efficiency compared to the newly generated CSN-W118A; in fact, the activity of CSN-VRE on low DA chitosan polymer was reduced by a factor of over 1100 compared to the activity of non-mutated CSN. Clearly, efficient large-scale production of pure D_4_ for downstream applications is best achieved using the new mutein CSN-W118A. Applying enzyme engineering allowed the production of D_4_ from low DA chitosan polymer as one of the main products in a batch process. The general approach for engineering CSN could potentially also be applied for enzymes from other glycoside hydrolase (GH) families if their active center residues are well-studied.

Single, pure fully defined chitosan oligomers such as the ones that can be produced using chitin deacetylases acting on, e.g., D_4_ or A_4_, bear a great potential when it comes to understanding the specific bioactivities of partially acetylated chitosans and deciphering their structure-function relationships. However, a large-scale application of single paCOS, e.g., in agriculture, is impractical because purifying them would be much too costly and time consuming. Instead, well-defined mixtures of paCOS containing the target oligomer(s) in known concentrations and being devoid of potentially inhibiting oligomers [9, 16] could be produced in a single step using chitosanases. To test whether the newly generated muteins are potentially suitable for such an approach, we analyzed hydrolysates produced using them for differences in their composition compared to those produced with CSN. The subsite specificities and preferences of CSN and its muteins, which directly affect the composition of the paCOS mixtures produced by them, were compared using chitosan polymer DA 30% as a substrate. Because chitosanases typically have strong preferences for GlcN units, the average DP of hydrolysates produced from medium DA chitosans is larger than the DP of those produced from low DA chitosan; importantly, both increased oligomer length and partial acetylation are frequently associated with stronger bioactivities towards plants. In this context, it was previously shown that to elicit paCOS-induced defense reactions in *Arabidopsis thaliana* associated with an oxidative burst, the paCOS must have a DP of six or higher and at least four GlcNAc units [16]. While the overall composition of oligomeric products concerning their DP and DA was rather similar for the wildtype and mutein enzymes, we found striking differences in their PA. Whether or not these changes will influence bioactivities will be the subject of further studies.

Both the CSN wildtype and its muteins belong to the same class of chitosanases (former class III or current class A), with an absolute specificity for GlcN at subsites (− 2) and (− 1) (Fig. 6). Also, only slight differences were detected between CSN and the muteins regarding subunit preferences at any of the subsites ranging from (− 3) to (+ 3). However, only a single DA substrate and a late time point were tested in this study, while a full assessment of subsite specificities and preferences requires testing of multiple conditions [22].

**Fig. 8:**
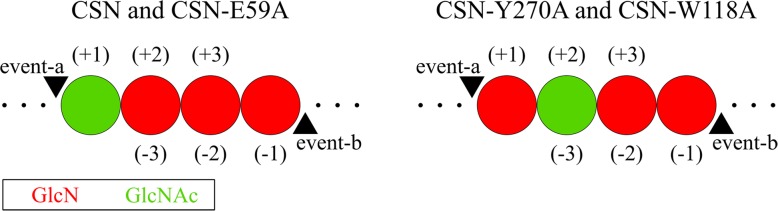
Visualization of the cleavage events a and b to produce A_1_D_3_. Pictured here are the most common PA of A_1_D_3_ found in the hydrolysates produced with CSN, CSN-Y270A, CSN-E59A, and CSN-W118A from Fig. 7. The positioning of the GlcN/GlcNAc units at the different subsites is shown. Also indicated by black triangles are the positions at which the two cleavage events must have occurred to produce the oligomer

Nevertheless, in spite of these minor differences in subsite preferences, we found distinct differences in the paCOS produced, particularly in their PA (Fig. 7), which might result in altered bioactivities of the hydrolysates. These structural differences were exemplified by the sequences of the monoacetylated tetramers A_1_D_3_, since these were present in all the hydrolysates and showed clear differences between the three muteins. Especially interesting in this context are CSN-E59A and CSN-W118A, since they almost exclusively produced ADDD or DADD, respectively. These differences between CSN and the muteins can be explained based on the mutations. The increased production of ADDD that was observed for CSN-E59A requires more frequent binding of an acetylated GlcNAc unit at the subsite (+ 1) (Fig. 8). Such an event is less likely to occur in CSN due to the predicted interaction of the negatively charged E59 with the positively charged amino group of a GlcN unit bound at subsite (+ 1) [36]. The substitution of E59 for alanine in CSN-E59A eliminates this effect, facilitating binding of a GlcNAc residue at subsite (+ 1). For both CSN-Y270A and CSN-W118A, more DADD was produced, as a result of a GlcNAc unit positioned at subsite (+ 2) or (− 3) during cleavage (Fig. 8). The substitution of Y270 for the smaller alanine widens the enzymatic cleft at the subsite (+ 2), allowing accommodation for the larger GlcNAc unit and facilitating its binding. The substitution of W118 for alanine could allow the D131 side chain located in close proximity to W118 at subsite (− 3) (Additional file 1: Figure S3) to move towards the space previously occupied by W118, which in CSN is predicted to form a stable hydrogen bond with the substrate [34]. As a result of replacing W118, steric hindrance at subsite (− 3) might no longer occur, and a GlcNAc unit might be more easily accepted there.

## Conclusion

In this work, rational protein engineering was used to generate three muteins of CSN from *Bacillus* sp. MN by impairing or abolishing stable substrate binding at specific subsites of the active center. The goals were twofold: First, we intended to increase from 4 to 5 the minimum DP CSN can cleave, thus allowing for the effective production of the fully deacetylated tetramer D_4_ in a batch process; second, we aimed to enable the generation of new hydrolysates with altered composition and, consequently, potentially altered bioactivities. Both goals were successfully achieved. CSN-E59A and CSN-W118A both showed reduced or missing activity on the tetramer compared to the wildtype enzyme. CSN-W118A was used to efficiently generate D_4_ from a chitosan polymer with very low DA, which was then purified to virtual homogeneity by size-exclusion chromatography. This tetramer, and the fully acetylated chitin tetramer A_4_ which can easily be produced from D_4_ using chemical N-acetylation, can now be used as a substrate for the generation of fully defined partially acetylated tetrameric COS using chitin deacetylases. Even though the subsite preferences of the three muteins were only slightly different from CSN, distinct differences in the PA of the paCOS products were found, showing that engineered chitosanases can yield novel paCOS mixtures potentially containing oligomers with specific bioactivities. Furthermore, a shifted substrate positioning in the active center of CSN-Y270A compared to the wildtype revealed that not all subsites are equally significant for substrate binding; such knowledge about the strength of the molecular interactions with the substrate at individual subsites can be valuable for further protein engineering.

## Methods

All chemicals were of analytical grade and purchased from Sigma-Aldrich (München, Germany) or Roth (Karlsruhe, Germany).

### Chitosans and COS

Chitosan polymer with DA 1.5%, DP 1300, and dispersity *Đ* of 1.8 (kindly provided by Mahtani Chitosan Pvt. Ltd., Veraval, India) was used directly or as the starting material for the preparation of chitosan polymer DA 30% by partial chemical *N*-acetylation under homogenous conditions according to Vachoud et al. [41]. The DA of the resulting chitosan was verified using ^1^H-NMR in acidic D_2_O at pD 3–4 [42]. DP and *Đ* were verified using high-performance size-exclusion chromatography (HP-SEC) with a differential refractometer and multi-angle laser light scattering [43]. Chitin and chitosan oligomers were either purchased from Carbosynth (Compton, UK) or produced enzymatically by hydrolysis of chitosan polymer DA 1.5% followed by separation through semi-preparative size-exclusion chromatography (SEC) (see section 1.7.1010) and mass spectrometric quantification (see section 1.7.8.2).

### Bacterial strains, vectors, and culture conditions

*Escherichia coli* DH5α was used as a host for recombinant plasmids, *E. coli* Rosetta 2 (DE3) [pLysSRARE2] (Merck KGaA, Darmstadt, Germany) was used for recombinant protein expression. The bacteria were incubated at 37 °C or 26 °C on either LB agar (Roth, Karlsruhe, Germany) or at 120–180 rpm in liquid LB medium (Roth, Karlsruhe, Germany) containing the appropriate antibiotics (100 μg/ml ampicillin or 100 μg/ml ampicillin and 34 μg/ml chloramphenicol) for plasmid stability. Stocks for long term storage at − 80 °C were prepared using 25% (*v*/v) glycerol and 0.5x LB medium.

### Generation of mutein-coding plasmids

The vector pET-22b(+)::*Strep*II-*csn* [33] was used as a template for the generation of all CSN muteins using a Rolling Circle PCR and subsequent ligation. Phusion Hot Start II High-Fidelity DNA Polymerase and T4 Rapid DNA Ligase (Fisher Scientific, Schwerte, Germany) were used according to the manufacturer’s instructions. The primers E59Afw (5′-Pho-TCCTTTTACGTAATAACCACCAGGTAAAG-3′) and E59Arev (5′-Pho-GCGATTACAGGTGATGCGGATGGG-3′) were used to introduce the mutation of E59 to A, resulting in the vector pET-22b(+)::*Strep*II-*csn-E59A*. The primers Y270Afw (5′-Pho-ATAATATGCATTTGTATATTCTGACTC-3′), Y270Arev (5′-Pho-GCGAATGCTAGTCGAGTACCTTTA-3′), W118Afw (5′-Pho-TCCCATTAAATTAGGATTTTGAGAG-3′) and W118Arev (5′-Pho-GCAGTTGTCGCAGATAGTAAAAAAGC-3′) were used analogously to create pET-22b(+)::*Strep*II-*csn-Y270A* and pET-22b(+)::*Strep*II-*csn-W118A*. The finished vectors were used for the transformation of *E. coli* DH5α as well as *E. coli* Rosetta 2 (DE3) [pLysSRARE2] and checked via sequencing. Primers were ordered from Eurofins MWG Operon (Eversberg, Germany). Sequencing was done by Microsynth Seqlab (Göttingen, Germany).

### Heterologous expression of CSN and the muteins

*E. coli* Rosetta 2 containing one of the respective plasmids was cultivated in 500 ml LB medium supplemented with solution M (50x, 1.25 M NaH_2_PO_4_, 1.25 M KH_2_PO_4_, 2.5 M NH_4_Cl, 0.25 M Na_2_SO_4_) and solution 5052 (50x, 25% [*v*/v] glycerol, 10% [*w*/*v*] α-lactose monohydrate, 2.5% [w/v] d-glucose) for autoinduction of the expression and antibiotics (34 μg/ml chloramphenicol and 100 μg/ml ampicillin) [44]. Incubation was done at 26 °C and 120 rpm for 48 h. Subsequent purification of the proteins was performed via *Strep*-tag II/*Strep*-Tactin affinity chromatography with 1 ml *Strep*-Tactin columns (Qiagen, Hilden, Germany) according to Nampally et al. [33], and the protein concentrations were measured with a Pierce™ BCA Protein Assay Kit (Fisher Scientific, Schwerte, Germany).

### SDS-PAGE and western blot

All samples were denatured in loading buffer (4x, 0.25 M Tris/HCl pH 6.8, 8% [*w*/*v*] SDS, 20% [w/v] glycerol, 0.04% [w/v] bromophenol blue, 0.4% [*v*/v] β-mercaptoethanol) for 10 min at 90 °C. The samples, Precision Plus Protein™ All Blue Prestained standard (Bio-Rad Laboratories, Hercules, USA), a 12.5% (w/v) polyacrylamide gel, and the appropriate buffer (0.025 M Tris, 0.192 M glycerol, 1% [w/v] SDS) were then used for SDS-PAGE according to Laemmli [45]. After separation, the proteins were either used for Coomassie staining (45% [v/v] methanol, 10% [v/v] acetic acid, 0.25% [w/v] CuSO_4_, 0.2% [w/v] Coomassie brilliant blue G250), or they were transferred to a nitrocellulose membrane (GE Healthcare Europe GmbH, Freiburg, Germany) using a semi-dry transfer procedure [46]. The membrane was incubated with 5% milk powder in TBS (10 mM Tris/HCl and 150 mM NaCl, pH 7.5) for 1 h. A *Strep-*Tactin horseradish peroxidase conjugate (IBA, Göttingen, Germany) was used according to the manufacturer’s instructions to detect all *Strep*-tag II fusion proteins.

### Enzymatic hydrolysis of chitosan polymers and oligomers

Sodium acetate buffer (40 mM, pH 6) was used for all enzymatic reactions and the temperature was controlled using a thermal mixer, except when producing D_4_ at a semitechnical scale. An incubation temperature of 37 °C was chosen when extensive hydrolysis was desired; for brief incubation times, 30 °C was used instead. Substrates were used at 1 mg/ml in the case of chitosan polymers and at 1 mM for D_4_ and D_5_. Exceptions to this were the enzymatic reactions to produce D_4_ at a semitechnical scale and those to determine kinetics. Enzyme concentrations varied as suitable concentrations were either determined experimentally or they were chosen based on the enzymes’ kinetic parameters. When preparing the samples for analysis by TLC, 0.05 μM of CSN, 0.3 μM of CSN-Y270A, 0.05 μM of CSN-E59A, and 2.5 μM of CSN-W118A were used and incubation was done at 37 °C for 16 h. The same enzyme concentrations were used when determining the subsite specificities of the enzymes. In this context, the hydrolysates were incubated at 37 °C for 48 h, then supplemented with an equal total amount of fresh enzyme and subsequently incubated for another 12 h to ensure that the end point of hydrolysis was reached, as verified via a reducing end assay (see section 1.7.7).

### Kinetics

Kinetics were determined on chitosan polymer DA 1.5% as well as on oligomers D_4_ and D_5_ at 30 °C using three independent batches of each enzyme and recording triplicates for each batch. The reaction velocities of the enzymes on the polymer were quantified using the reducing end assay (see section 1.7.7) to determine how many cleavage events occurred over time. The reactions were always incubated for 10 min (except for 30 min in the case of CSN-W118A) and the increase in reducing ends was determined between an early (t_0_) and a late timepoint (t_10_/t_30_). The tested substrate concentrations ranged from 0.15–2.5 mg/ml chitosan polymer DA 1.5%, and enzymes CSN, CSN-Y270A, CSN-E59A, and CSN-W118A were used at 0.05 μM, 0.3 μM, 0.05 μM, and 2.5 μM, respectively. A quantification of the enzymatic activity on D_4_ and D_5_ was achieved by using UHPLC-ELSD-ESI-MS^1^ measurements and external oligomer standards (see section 1.7.8.1) to determine the increase of the relevant oligomers between two different time points. The reaction velocities were linear for the chosen substrate concentrations, enzyme concentrations, and incubation times. D_4_ and D_5_ were used at 0.01–2 mM, the reaction time was set to 10 min (except for 30 min in the cases of CSN-E59A with D_4_ and CSN-W118A with D_5_), and the enzymes were used at concentrations of 0.04 μM (CSN for D_4_), 0.02 μM (CSN for D_5_), 0.023 μM (CSN-Y270A for D_4_), 0.168 μM (CSN-Y270A for D_5_), 0.1 μM (CSN-E59A for D_4_), 0.2 μM (CSN-E59A for D_5_), and 1.0 μM (CSN-W118A). For further analysis, the program Origin 8 (OriginLab Corporation, USA) was used. The reaction velocities were plotted against the corresponding substrate concentrations and a Hill fit was applied to determine kinetic parameters. The number of reducing ends formed for D_4_ was calculated by adding the amount of D_2_ divided by the factor 2 (cleavage of D_4_ into D_2_ and D_2_) and the amount of D_3_ (cleavage of D_4_ into D_3_ and D_1_). When D_5_ was used as the substrate, the amounts of D_2_ and D_3_ were added and halved (cleavage of D_5_ into D_2_ and D_3_) and added to the amount of D_4_ (cleavage of D_5_ into D_1_ and D_4_). This was done under the assumption that any D_4_ formed from D_5_ would not be used as a substrate by the enzymes during the 10–30 min incubation time because D_5_ would still be the predominant oligomer in the reactions and is a preferred substrate for all enzymes used.

#### Determination of the cleavage position when digesting D_4_ and D_5_

D_4_ and D_5_ were labeled with ^18^O at the reducing end using H_2_^18^O (see section 1.7.8.2). Since the ^18^O is gradually exchanged for ^16^O once the substrates come into contact with H_2_^16^O, the subsequent enzymatic reactions and UHPLC-ELSD-ESI-MS^1^ measurements were kept as brief as possible. The enzymes were used at different concentrations (0.02 μM of CSN for D_4_ and 0.16 μM for D_5_, 0.035 μM of CSN-Y270A for D_4_ and 0.42 μM for D_5_, 0.33 μM of CSN-E59A for D_5_, 5 μM of CSN-W118A for D_5_) and were incubated with the substrates for 10 min at 30 °C, immediately followed by the measurement. The oligomers were then quantified using external oligomer standards (see section 1.7.8.1).

#### Production of D_4_ at a semitechnical scale

One g of chitosan polymer DA 1.5% (5 mg/ml) and the mutein CSN-W118A (0.367 μM) were used to produce D_4_ at a semitechnical scale. The enzyme was purified as described before, but without the use of TEA to avoid contamination of the product. The enzymatic reaction was performed in 2 mM ammonium formiate buffer pH 5.5 and was stirred for 51.5 h at 37 °C. A water bath was used to control the temperature. During the reaction, the progress of the hydrolysis was monitored using UHPLC-ELSD-ESI-MS^1^-measurements. When almost no pentamer or larger oligomers were detectable anymore, the reaction was ended by freezing the solution. The D_4_ was separated from other oligomers using SEC and then quantified as well as checked for its purity using reducing end assay (see section 1.7.7) and ESI-MS^1^ with internal R* standards (see section 1.7.8.2). Analogously, another D_4_ production procedure was performed using mutein CSN-E59A, but at a concentration of 0.2 μM, and the incubation time was limited to 20.66 h.

### Reducing end assay

For the determination of kinetics on chitosan polymer DA 1.5%, a reducing end assay as described by Horn and Eijsink [47] was used to quantify the newly formed reducing ends over time. The enzymatic reactions were stopped by adding 0.5 M NaOH. The amount of reducing ends in the sample was determined by measuring the absorption at 620 nm with a Multiskan® GO microplate spectrophotometer (Fisher Scientific, Schwerte, Germany) and then comparing it to that of *d*-glucosamine standards (0.05–1.5 mM).

### UHPLC-ELSD-ESI-MS^n^ analysis

Chitosan oligomers were separated using a Dionex Ultimate 3000RS UHPLC system (Thermo Fisher Scientific, Waltham, USA) coupled to an evaporative light scattering detector (Model Sedex 90LT, Sedere, Alfortville Cedex, France) and an ESI-MS^n^-detector (amaZon speed, Bruker, Bremen, Germany). Separation of the oligomers was achieved by hydrophilic interaction liquid chromatography (HILIC) using an Acquity UHPLC BEH Amide column (1.7 μm, 2.1 mm × 150 mm; Waters Corporation, Milford, USA) in combination with a VanGuard pre-column (1.7 μm, 2.1 mm × 5 mm; Waters Corporation, Milford, USA). The samples were split between ELSD and ESI-MS^n^ detectors using a 1:1 splitter (Accurate, Dionex Corporation, Sunnyvale, USA). All of the used UHPLC-ELSD-ESI-MS^n^ methods were based on the ones described by Cord-Landwehr et al. [17]. The injection volume was always 1 μl for undiluted samples and 2 μl for samples that were diluted with equal parts of 0.5 M NaOH to stop an enzymatic reaction. Data Analysis 4.1 software (Bruker, Bremen, Germany) was used for analysis of the results.

#### Quantification of chitosan oligomers using external standards

For determination of kinetics using D_4_ and D_5_ as well as for analysis of the substrate positioning of those oligomers, UHPLC-ELSD-ESI-MS^1^ methods (gradient elution profile in Additional file 1: Table S1) and external oligomer standards were used. Standards were measured using the same method and buffers as in the respective experiments using D_2_, D_3_, and D_4_ (0.001–1 mM). D_1_ was not measured since it ionizes very poorly using the described setup and the amounts of D_1_ present in the samples were deduced from the measured amounts of the other oligomers.

#### Quantitative sequencing

For determination of the enzymes’ subsite specificities, quantitative sequencing was used as described by Cord-Landwehr et al. [17]. The method involves quantitative UHPLC-ELSD-ESI-MS^1^ measurements for quantification of the oligomers and determination of DP and DA combined with UHPLC-ELSD-ESI-MS^2^ measurements to determine the pattern of acetylation. The oligomers in the produced hydrolysates were deutero-*N*-acetylated with [^2^H_6_]-acetic anhydride (Sigma-Aldrich, St. Louis, USA). This caused oligomers with the same DP, but different DAs to elute at the same time and allowed the use of a single internal standard for each DP. As standards, GlcN_2–6_ oligomers were *N*-acetylated using [^13^C_4_, ^2^H_6_]-acetic anhydride (Sigma-Aldrich, St. Louis, USA); these are referred to as R*_2–6_ standards. The deutero-*N*-acetylated samples and the R*_2–6_ standards were mixed, dried in vacuo, and resolved in water and then, 2 μl containing 2 μg of the oligomers and 75 ng of each of the standards were analyzed via LC-MS^1^ (gradient elution profile in Additional file 1: Table S1). The target mass was changed over time, adjusting it to the eluting oligomers. This was done to optimize ion transmission and detection of the products. To allow quantitative pattern determination, the deutero-*N*-acetylated hydrolysates were dried in vacuo and then labeled at the reducing ends with ^18^O using H_2_^18^O (euriso-top, Saint-Aubin, France). One μg of each hydrolysate was then analyzed via LC-MS^2^ (gradient elution profile in Additional file 1: Table S1). The results of the LC-MS^1^ and LC-MS^2^ measurements were then combined and evaluated according to Cord-Landwehr et al. [17].

#### Thin layer chromatography (TLC)

Silica plate high performance thin-layer chromatography (HP-TLC) was used to qualitatively analyze chitosan oligomers produced by enzymatic hydrolysis. Aliquots (25 μg) of the oligomers in 10 μl H_2_O and a GlcN_1–6_ standard (12 μg of each oligomer) were sprayed on a TLC plate coated with silica gel (Merck KGaA, Darmstadt, Germany) using an Automatic TLC sampler 4 (CAMAG, Switzerland). For the subsequent separation, a 5:4:2:1 (*v*/v/v/v) mixture of *n*-butanol, methanol, 25% (v/v) ammonia, and H_2_O was used. The TLC plate was then briefly dipped into 30% (v/v) ammonium bisulfate and heated using a hot air gun until the separated products were visible [48].

#### Size-exclusion chromatography (SEC)

Preparative separation of chitosan oligomers was achieved by size-exclusion chromatography using a SECcurity GPC System (PSS Polymer Standards Service, Mainz, Germany), three serial HiLoad 26/600 Superdex 30 prep grade columns (GE Healthcare Europe GmbH, Freiburg, Germany), and an Agilent 1200 series refractive index detector (Agilent Technologies, Santa Clara, USA). For data recording, the WinGPC UniChrom software (PSS Polymer Standards Service, Mainz, Germany) was used. Aliquots (100 mg each) of an oligomer mixture were dissolved in ammonium acetate buffer (0.15 M, pH 4.5) and separated using the same buffer as the mobile phase with a flow rate of 0.6 ml/min. The oligomers were collected in 3 ml fractions, analyzed via UHPLC-ELSD-LC-MS^1^, and pooled accordingly. Twice the equimolar amount of HCl relative to the number of GlcN subunits was added to stabilize the oligomers. Ammonium acetate was removed by repeated freeze drying and dissolving in H_2_O.

#### Molecular modeling and docking studies

The model of CSN with a docked GlcN hexamer (Fig. 1, Additional file 1: Figure S3) was generated using SWISS-MODEL [49, 50] using the crystal structure of *Bacillus* sp. K17 chitosanase (PDB: 1V5C, amino acid sequence identity: 97.47%) as a template. Post refinement including energy minimization stereo-chemical correction was performed using the KoBaMIN server [51]. MolProbity [52] was used to assess the geometric accuracy of the refined model. The substrate was built with the GLYCAM webserver [53], charges were assigned, hydrogens were added, and nonpolar hydrogens were merged. Docking was performed using AutoDock 4.2. The inbuilt *autogrid* function in AutoDock was used to generate a grid map around the enzyme’s active center. The Lamarckian Genetic Algorithm with default parameters was applied and 100 conformations were generated for the substrate. The binding energy of the docked substrate was evaluated using the *autoscorer* function in AutoDock and the best results were visualized using PYMOL (The PYMOL Molecular Graphics System, Version 1.8 Schrödinger, LLC).

## Additional file

Additional file 1: **Figure S1.** Western blots of CSN, CSN-E59A, CSN-Y270A, and CSN-W118A. **Figure S2.** Quantification of A_1_D_3_. **Figure S3.** Selected CSN residues with a predicted or experimentally verified function in substrate binding. **Figure S4.** Comparison of expected and determined productive substrate positionings of D_5_ and D_4_ in CSN and the muteins. **Table S1.** Gradient elution profiles, temperatures, and flow rates of the employed UHPLC-MS^n^ methods. (DOCX 2671 kb)

## Abbreviations

A_n_: n-mer of *N*-acetyl-d-glucosamine
CSN: Chitosanase *Bsp*CsnMN from *Bacillus* sp. MN
DA: Degree of acetylation
D_n_: n-mer of d-glucosamine
DP: Degree of polymerization
GH: Glycoside hydrolase
GlcN: d-glucosamine
GlcNAc: *N*-acetyl-d-glucosamine
HILIC: Hydrophilic interaction liquid chromatography
k_cat_: Catalytic rate constant
PA: Pattern of acetylation
paCOS: Partially acetylated chito-oligosaccharides
SEC: Size-exclusion chromatography
TLC: Thin layer chromatography
UF: Ultrafiltration
UHPLC-ELSD-ESI-MS: Ultra high performance liquid chromatography - evaporative light scattering detection - electrospray ionization - mass spectrometry
