# Supplementary material for: Rational protein design of Bacillus sp. MN chitosanase for altered substrate binding and production of specific chitosan oligomers
Source: J Biol Eng. 2019 Mar 12;13:23. doi: 10.1186/s13036-019-0152-9 (PMC6419424; doi:10.1186/s13036-019-0152-9)
Supplement: Supplementary file 1 — Figure S1. Western blots of CSN, CSN-E59A, CSN-Y270A, and CSN-W118A. Figure S2. Quantification of A1D3. Figure S3. Selected CSN residues with a predicted or experimentally verified function in substrate binding. Figure S4. Comparison of expected and determined productive substrate positionings of D5 and D4 in CSN and the muteins. Table S1. Gradient elution profiles, temperatures, and flow rates of the employed UHPLC-MSn methods. (DOCX 2671 kb) [file 13036_2019_152_MOESM1_ESM.docx]

Additional file 1:


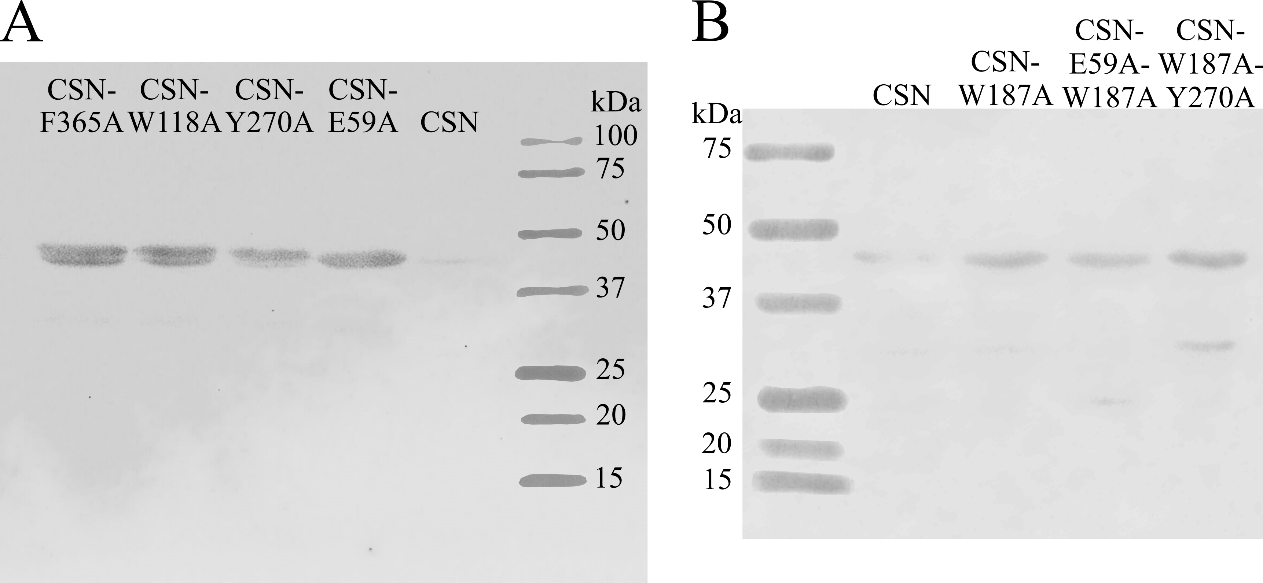


Figure S1: Western blots of CSN, CSN-E59A, CSN-Y270A, and CSN-W118A. Six µg of purified protein were loaded to the gel and transferred to a nitrocellulose membrane after electrophoresis. The expected size for CSN and all muteins is 48 kDa. Precision Plus Protein™ All Blue Prestained standard was used and the bands were inserted into the picture by merging a white light photograph and a chemiluminescence photograph.


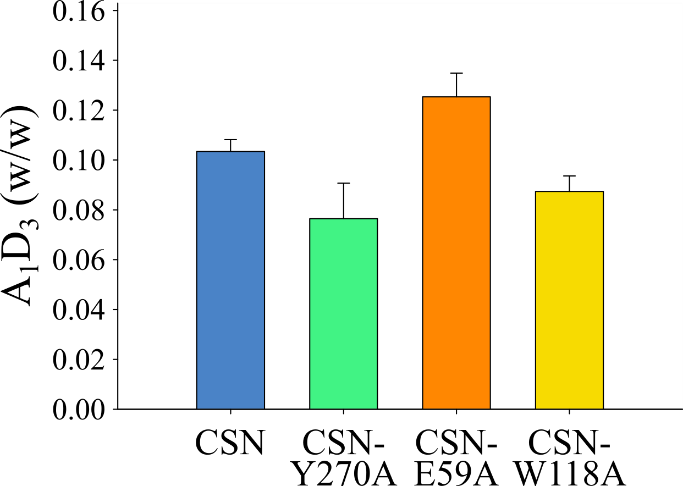


Figure S2: Quantification of A_1_D_3_. Amount of the monoacetylated chitosan tetramer A_1_D_3_ in the hydrolysates used to determine the frequency of each A_1_D_3_ pattern for CSN, CSN-Y270A, CSN-E59A, and CSN-W118A (see Figure 6). The experiments were performed as triplicates with one batch of each enzyme. Data given are the mean values and standard deviation are indicated.


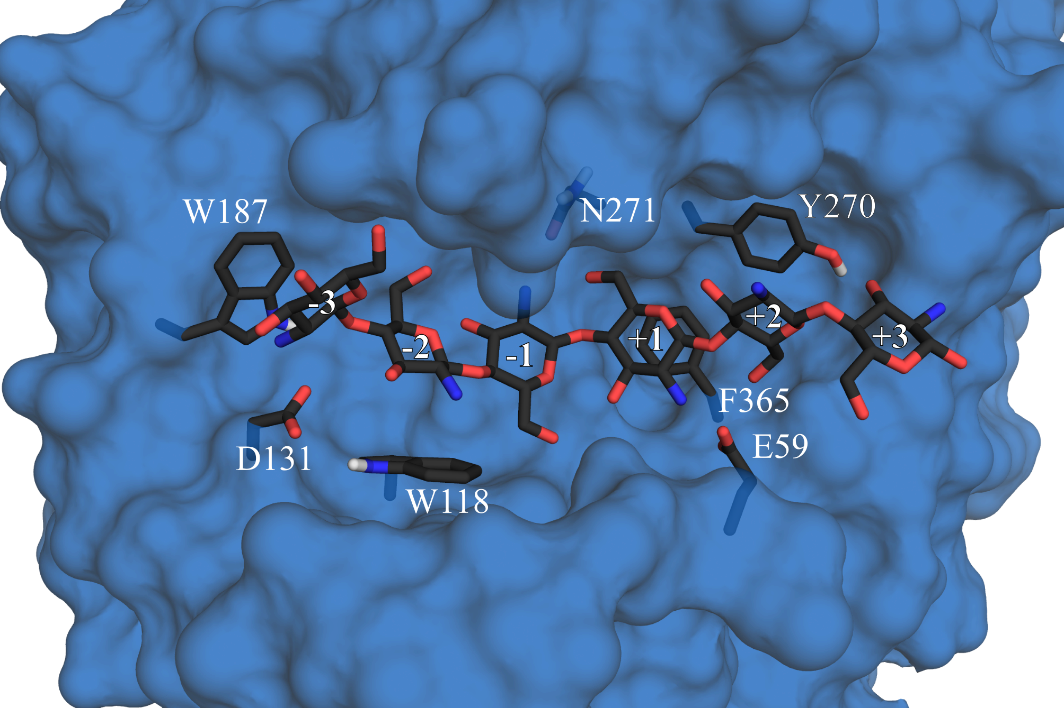


Figure S3: Selected CSN residues with a predicted or experimentally verified function in substrate binding. A three-dimensional homology-based model of CSN, illustrating the side chain positions of the amino acids E59, W118, D131, W187, Y270, N271, and F365 relative to the docked substrate D_6_. The enzyme surface without the side chains of the listed amino acids is pictured in blue, the labeled amino acid side chains emerging from the surface and D_6_ are colored by element. Experimental verifications were done as part of this study, *in silico* predictions are based on Singh et al. (2019) .


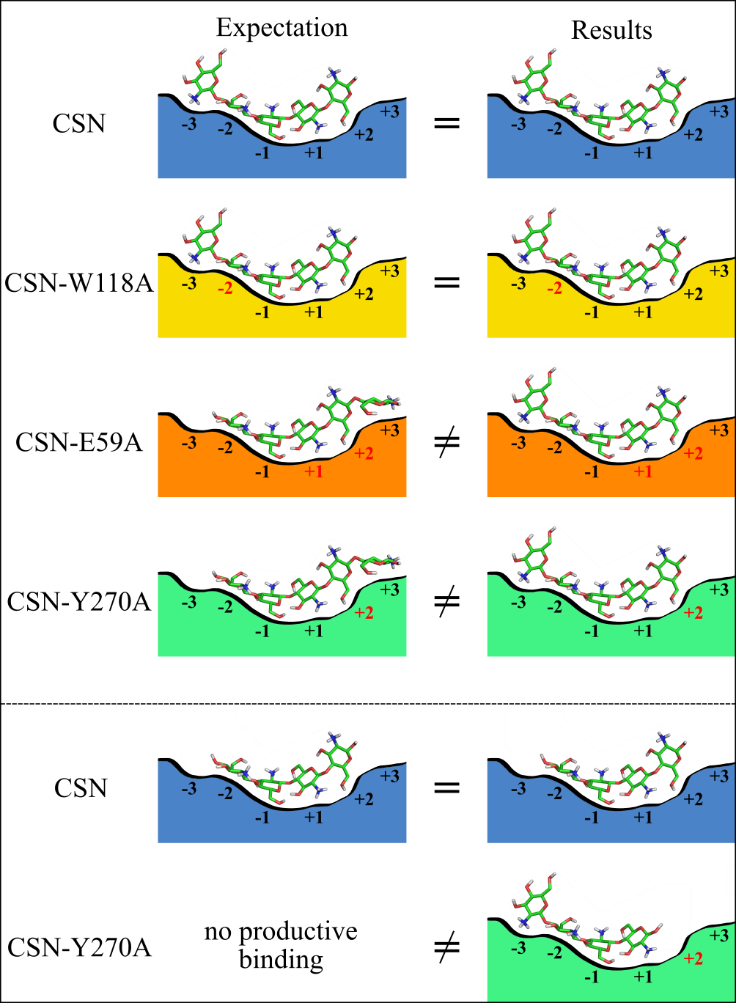


Figure S4: Comparison of expected and determined productive substrate positionings of D_5_ and D_4_ in CSN and the muteins. The initially expected and the determined most frequent substrate positionings of D_5_ and D_4_ in the active centers of CSN and the muteins CSN-W118A, CSN-E59A, and CSN-Y270A are illustrated schematically. The numbers indicating the mutated subsites are printed in red. The positions are based on results shown in Figure 4.

Table S1: Gradient elution profiles, temperatures, and flow rates of the employed UHPLC-MS^n^ methods.

| **Method for** | **Time [min]** | **Gradient** | **Eluent** | **Temperature and flowrate** |
| --- | --- | --- | --- | --- |
| kinetics | 0–0.8 | isocratic | 30% eluent B | 70°C and  0.8 ml/min |
|  | 0.8–2.633 | linear | 30–85% eluent B |  |
|  | 2.633-2.833 | linear | 85–30% eluent B |  |
|  | 2.833-3.633 | isocratic | 30% eluent B |  |
| ^18^O-labeling | 0–5 | linear | 40–90% eluent B | 35°C and  0.4 ml/min |
|  | 5–5.5 | linear | 90–40% eluent B |  |
|  | 5.5–7 | isocratic | 40% eluent B |  |
| Quantitative  sequencing  (MS^1^ and MS^2^) | 0–3 | isocratic | 0% eluent B | 35°C and  0.4 ml/min |
|  | 3–23 | linear | 0–30% eluent B |  |
|  | 23–25 | linear | 30–75% eluent B |  |
|  | 25–26 | isocratic | 75% eluent B |  |
|  | 26–27 | linear | 75–0% eluent B |  |
|  | 27–30 | isocratic | 0% eluent B |  |
